# DeepGeSeq: deep learning library for genomic sequence modeling and analysis

**DOI:** 10.1093/bioinformatics/btag584

**Published:** 2026-08-04

**Authors:** Jiaqi Li

**Affiliations:** Centre for Evolutionary and Organismal Biology, Liangzhu Laboratory, Zhejiang University School of Medicine, Hangzhou 310058, China

## Abstract

**Motivation:**

Deep learning methods have demonstrated significant potential in genomics, enabling broad applications such as sequence activity prediction, regulatory rule identification, and variant effect quantification. However, their widespread adoption is often hindered by the steep computational learning curve required for model construction, training, and downstream biological interpretation. Here, we introduce DeepGeSeq, a user-friendly Deep-learning library tailored for Genomic Sequence modeling and analysis.

**Results:**

By integrating state-of-the-art architectural modules, DeepGeSeq streamlines the entire deep learning workflow, requiring minimal user input via a simple configuration file and an intuitive agentic skill. We comprehensively validate the efficacy of DeepGeSeq through diverse case studies, encompassing pipeline verification using synthetic datasets, the reproduction and application of established models, and model fine-tuning coupled with biological interpretation on user-defined data. Furthermore, we demonstrate DeepGeSeq’s versatility in domain-specific applications, including single-cell ATAC-seq modeling for cell-type clustering, and MPRA data modeling coupled with in silico saturation mutagenesis to dissect cis-regulatory elements. Ultimately, DeepGeSeq bridges the gap between computational complexity and biological discovery, providing an accessible resource that facilitates the development and broad application of deep learning methods in genomics research.

**Availability and implementation:**

https://github.com/JiaqiLi1024/DeepGeSeq.

## 1 Introduction

Deep learning-based methods have emerged as powerful tools for addressing fundamental challenges in genomics, enabling the precise modeling of complex biological processes (Sasse *et al*. 2024). A wide range of deep learning models has been developed to decode regulatory information directly from genomic sequences. For example, models such as DeepSEA (Zhou and Troyanskaya 2015), Basset (Kelley *et al*. 2016), AI-TAC (Maslova *et al.* 2020), and DanQ (Quang and Xie 2016) have leveraged large-scale chromatin-profiling datasets, including transcription factor binding, histone marks, and chromatin accessibility, to uncover regulatory patterns embedded in DNA sequences. In the context of transcriptional regulation, extensive efforts have focused on bridging regulatory sequences with gene expression outcomes. These approaches range from predicting gene expression solely from DNA sequences [e.g. Xpresso (Agarwal and Shendure 2020) and Nvwa (Li *et al.* 2022)], to integrating chromatin profiles [e.g. Enformer (Avsec *et al.* 2021), Basenji (Kelley *et al.* 2018), ChromBPNet (Pampari *et al.* 2025), and Borzoi (Linder *et al.* 2025)], and utilizing previously predicted regulatory features for hierarchical modeling [e.g. Expecto (Zhou *et al.* 2018), SVEN (Wang *et al*. 2024), and EPInformer (Lin *et al*. 2024)]. Beyond predictive modeling, sequence-based frameworks have demonstrated significant value in downstream applications. Notable examples include identifying functional regulatory elements [e.g. Basset, AI-TAC, and DeepFlyBrain (Janssens *et al.* 2022)], quantifying the effects of genetic variants and their implications for disease (e.g. DeepSEA, Expecto, and Enformer), and exploring cross-species evolutionary patterns [e.g. TACIT (Kaplow *et al.* 2023)]. Conversely, foundation models [e.g. DNABERT (Zhou *et al.* 2024), Evo (Brixi *et al.* 2026), Nucleotide Transformer (Dalla-Torre *et al.* 2025)] expand the field by uncovering regulatory grammar and evolutionary constraints from multispecies or genome-scale data. Collectively, these advances underscore the transformative potential of deep learning in decoding the regulatory genome and unlocking novel avenues for functional genomics research.

However, the development of deep neural networks frequently demands substantial effort across model construction, training, interpretation, and deployment. Crucially, a major challenge lies in the fact that incorrect inputs or flawed procedures can lead to erroneous predictions and interpretations, often without explicit error messages to alert users to the underlying issues. Recognizing these bottlenecks, researchers have developed unified software frameworks aimed at streamlining those processes and mitigating such risks. Nevertheless, existing frameworks still exhibit notable limitations in their scope. For example, while Kipoi (Avsec *et al.* 2019) primarily focuses on the distribution of pre-trained models, it lacks an integrated end-to-end workflow. Selene (Chen *et al.* 2019) and Janggu (Kopp *et al.* 2020) provide comprehensive deep-learning pipelines for model training, yet their utility in model interpretation remains limited. Even the most comprehensive framework, such as gReLU (Lal *et al.* 2024) and EUGENe (Klie *et al.* 2023), which provide biologically intuitive workflows, falls short in native support for emerging downstream analyses and broader community accessibility. Despite these advances, there remains a pressing need for a user-friendly yet comprehensive library that integrates state-of-the-art models with versatile workflows for both biologists and bioinformaticians. Such a tool would bridge the gap between technical complexity and practical usability, thereby facilitating broader adoption and more effective application in biological research.

Here, we present DeepGeSeq, a deep learning library for genomic sequence modeling and analysis. DeepGeSeq supports biologists and bioinformaticians by providing state-of-the-art deep learning modules, model development, automatic architecture searching, and model training in a user-friendly manner. To simplify the application of genomic sequence models, DeepGeSeq directly takes BED and bigWig files from upstream pipelines, produces enriched sequence motifs as the output files, and calculates the scores of corresponding sequences or mutations for further analysis, which are of particular interest to biologists. To demonstrate advanced usage, several case studies were performed to validate and illustrate the overall workflow of DeepGeSeq. First, we (i) validated the entire pipeline on a synthetic dataset, then (ii) reproduced a previously reported model on user-defined data, and (iii) utilized the trained model for real-world genomic analysis. We further showcased DeepGeSeq’s utility in (iv) single-cell ATAC data modeling and dimensionality reduction for cell-type clustering, and (v) MPRA data modeling and saturation mutagenesis for cis-regulatory element analysis. All the usage examples and case studies are provided as the tutorials for DeepGeSeq at https://github.com/JiaqiLi1024/DeepGeSeq.

## 2 Methods

### 2.1 Overview of the DeepGeSeq package

The DeepGeSeq packages were implemented using PyTorch, one of the most popular deep learning backends. DeepGeSeq comprises three main components: a python library with modules for developing neural networks; a suite of tools for training, evaluating, and interpreting the sequence-based model, and a user-friendly interface for executing experiments via a JSON configuration file.

The DeepGeSeq IO/Data introduce built-in data preprocessing modules capable of seamlessly handling multi-format inputs, including FASTA, BED, bigWig, and VCF files. The DeepGeSeq Module and Model libraries define the foundational elements for building neural network architectures. The Module library contains basic components, including convolutional, recurrent, and linear layers, alongside advanced mechanisms such as residual blocks and attention modules (e.g. Multi-Head Attention and Convolutional Block Attention Module). The Model library implements a diverse array of model architectures, ranging from standard CNN, RNN, and Transformer to widely used published sequence-based architectures (e.g. DeepSEA, Beluga, DanQ, Basset, ChromBPNet, Borzoi, Enformer) and genomic language models (e.g. Evo, DNABERT, and NT). Furthermore, the Extension module provides utilities for loading and instantiating custom models.

The DeepGeSeq Trainer, Evaluator, and Explainer provide comprehensive support for the model lifecycle. Instantiated models are automatically trained on the input dataset using the Trainer, which leverages the PyTorch backend for GPU acceleration. During training, users can monitor validation metrics and losses, with enhanced visualization available via TensorBoard integration. Following model training, the Evaluator enables users to assess the model on the held-out test set and calculate the predictive metrics across different tasks. The Explainer supports feature-map and gradient-based interpretation methods, along with advanced attribution algorithms like DeepLIFT and DeepSHAP. Specifically, the Motif module interprets learned sequence patterns by converting and visualizing the model filters into position weighted matrices (PWMs) for downstream analysis, such as TomTom and HOMER. Additionally, TF-MoDISco is integrated for attribution-based de novo motif discovery. The Influence module performs in silico perturbation on the sequence input and feature maps to calculate their influence on each task. Building upon the predictive model, DeepGeSeq also features an advanced sequence design module, enabling users to perform exploratory in silico sequence generation and optimization using gradient ascent and greedy mutagenesis strategies.

The top-level DeepGeSeq interface and config module provide high-level functionality for users to execute experiments. A JSON file configures the parameters of experiment modes, model architectures, and datasets. Users can select several modes to automate the respective workflow on the configured dataset, including “train,” “evaluate,” and “explain.” A minimal DeepGeSeq experiment can be entirely compiled within a config.json file, allowing users to tailor data, model, modes to suit the specific training task. To further lower the technical barrier, DeepGeSeq now incorporates an agentic skill, empowering biologists to set configurations and interact with the deep learning framework entirely through natural language. We recommend starting from one of the example training files in the GitHub tutorials.

### 2.2 General steps to reproduce the case studies

In this section, we outline the general workflow for reproducing the case studies. Although each case study involves unique biological significance, datasets, and analytical goals, the underlying model components share a high degree of universal applicability. Here, we provide a shared methodological framework for model architecture, training, evaluation, and interpretation. While these general steps provide a standardized foundation for reproducing and extending our analyses, the unique methodologies and specific operations tailored to each case study are elaborated in the subsequent sections.

#### 2.2.1 Baseline model architecture

The baseline model consists of two primary blocks, with the encoder of a convolutional neural network (CNN) and the decoder of a fully connected feed-forward network (FFN). The encoder block contains two convolutional layers. The first convolutional layer applies a set of motif detectors that scan along the sequence, transforming the genomic input into feature maps. Each motif detector is represented by a 4×*m* weight matrix (*m* = 15 in baseline model), which can be conceptualized as a position weight matrix (PWM) of length m. In the second convolutional layer, each weight matrix captures the degree of interaction between these motifs across the feature maps generated by the previous layer. Following the convolutional operations, the outputs are transformed by the rectified linear activation function (ReLU), which clamps negative values to zero. A global average pooling (GAP) layer then computes the average of these rectified outputs across the entire sequence. The decoder block consists of two linear transformations separated by a ReLU activation. Finally, the pooled features are fed into the FFN decoder to predict the cell expression state, with the final prediction scaled into an expression probability using a sigmoid function.

#### 2.2.2 DeepSEA model architecture

In Case Studies 2 and 3, we utilized the DeepSEA architecture, adopting the detailed parameter settings directly from the original publication. The architecture balances feature extraction through convolutional layers, dimensionality reduction via pooling, and robustness through strategic dropout rates. Briefly, the model architecture consists of three convolutional layers (320, 480, and 960 filters, respectively) with the window size of 8 and the stride size of 1, interspersed with two max-pooling layers (window size 4, stride 4). The extracted features are integrated by a fully connected layer with 919 units, corresponding to specific genomic features, and scaled into prediction probabilities using a final sigmoid activation function.

#### 2.2.3 Basset model architecture

In Case Studies 3, we utilized the Basset architecture, adopting the parameter settings directly from the mouse sci-ATAC publication. Compared to DeepSEA, Basset employs increased filter numbers and sizes to capture a broader range of sequence features. The architecture is structured as follows: three convolutional layers (600 filters, window size 19 in Layer 1; 200 filters, window size 11 in Layer 2; 200 filters, window size 7 in Layer 3; all with a stride of 1), followed by max-pooling (window size 3, stride 3 in Layer 1; window size 4, stride 4 in Layers 2 and 3). The extracted features are then processed by two fully connected layers (1000 units each) separated by a ReLU activation, with 30% dropout applied to both to prevent overfitting. Final predictions are scaled to probabilities via a sigmoid output layer.

#### 2.2.4 Training of models

At the beginning of training, all parameters were initialized using “Kaiming initialization.” A mini-batch of randomly selected input sequences (*N* = 128) was fed through the network to generate predictions. The objective function was minimized by standard backpropagation, and the learnable parameters were updated using the Adam optimizer. To avoid overfitting, early stopping was applied once the model converged on the validation set. All models were implemented using DeepGeSeq v1.0, PyTorch (https://pytorch.org) 2.8, CUDA 12.8, the Anaconda3 distribution of Python and trained on computer cluster equipped four NVIDIA A100 and GeForce RTX 5090 Tensor Core GPUs.

#### 2.2.5 Evaluation of prediction performance

The prediction performance of the model was evaluated on a held-out test set, which remained strictly isolated during the training phase to prevent information leakage. For each task, we computed several standard metrics to evaluate the predictions, including Pearson correlation coefficient (PCC), Spearman correlation coefficient (SCC), Area Under the Receiver Operating Characteristic Curve (AUROC), and Area Under the Precision-Recall Curve (AUPR). Specifically, PCC and SCC was utilized to assess regression performance, while AUROC and AUPR were employed to evaluate classification performance.

#### 2.2.6 Model interpretation

We applied a feature-map-based method within DeepGeSeq to interpret the model parameters. Similar to the node-based strategy in Basset7, each first-layer convolution filters was extracted and converted into a PWM by counting nucleotide occurrences within the feature maps. We utilized an activation threshold of 0.9, which means only nucleotide sequences achieving at least 90% of a filter’s maximum activation value were counted in the PWM. Finally, we calculated the information content (IC) for each resulting PWM.

### 2.3 Case study #1

To simplify and validate the representation learning of CNN, we generated a synthetic dataset containing with known ground truth motifs29. Specifically, these motifs were embedded into random synthetic DNA sequences to construct a multi-task classification dataset. Following a procedure similar to a previous study14, we generated 500-nt random DNA sequences and randomly embedded selected TF PWMs into them (forward and reverse complements orientations). We acquired a pool of 4 PWMs corresponding to 4 unique TFs from the JASPAR database: CEBPB, CTCF, TP53 and SRF. A corresponding label vector were generated for each sequence to indicate the presence of specific motif. In total, we generated 25 000 sequence-label pairs, and randomly partitioned the synthetic dataset into training, validation, and test sets.

For the general experimental settings, we utilized the baseline model architecture. The training, evaluation, and interpretation procedures were identical to those described previously.

### 2.4 Case study #2

In this case study, we utilized the DeepSEA dataset, comprising 919 chromatin features, downloaded from the original publication. For the general experimental settings, we employed the DeepSEA model architecture. The training and evaluation procedures were identical to those described previously.

We then applied the reproduced DeepSEA model to analyse the variant effects of single-nucleotide polymorphisms (SNPs) derived from the International Genomics of Alzheimer’s Project meta-analysis study. To evaluate the model’s ability to prioritize functionally relevant variants, SNPs were categorized into two groups based on their association with Alzheimer’s disease: a significant variant set (*P* < .05; *n* = 4 22 398) and a non-significant background set (*P* > .50; *n* = 38 42 725), extracted from a total of 1 06 87 126 genetic variants. We then performed variant effect prediction to quantify the differential impact of alternative alleles relative to native reference sequences, leveraging the sequence-based model’s capacity to predict chromatin effects of sequence alterations. For the statistical analysis, we conducted one-sided Wilcoxon rank-sum tests across all 919 output tasks to compare the predicted effect distributions between the significant and non-significant variant sets. Adjusted *P* values were subsequently calculated using the Benjamini–Hochberg procedure to control the false discovery rate and ensure robust correction for multiple testing.

### 2.5 Case study #3

In this case study, a mouse single-cell ATAC dataset was downloaded from a previously published sci-ATAC study26. For the general experimental settings, we employed both the Basset and DeepSEA model architectures. Training, evaluation, and interpretation procedures were identical to those described above.

To annotate and analyse the motifs learned by the convolutional neural network (CNN) models, we converted the filter weights of the first convolutional layer into position weight matrices (PWMs) using both feature-map-based and gradient-based methods. Specifically, consistent with the hyperparameters defined in the original studies, we extracted 600 motifs with a length of 19 from the Basset model and 320 motifs with a length of 8 from the DeepSEA model. We then utilized the TomTom algorithm to evaluate these learned motifs by identifying significant matches using a *P*-value cutoff of .05. Using this approach, we first compared the motifs extracted from the reproduced models against those reported in the original publications. Subsequently, we annotated the derived motifs from both models by assigning them to known transcription factor binding sites (TFBSs) curated in the CIS-BP and JASPAR databases using the TomTom algorithm.

### 2.6 Case study #4

In this case study, the single-cell chromatin accessibility dataset was obtained from Buenrostro. We adopted the scBasset processing method to generate the input sequences and corresponding labels, subsequently partitioning the data into training and test sets at a 9:1 ratio. For the general experimental settings, we employed the baseline model architecture. The training procedures were identical to those described previously.

To evaluate the predicted cell relationship within the test set, we calculated the average AUROC and AUPR for each cell lineage. To evaluate the quality of cell embedding, the weights of the final linear layer were projected into a t-SNE embedding space using standard procedures in Scanpy. Adjusted mutual information (AMI) was then applied to quantitatively measure the clustering similarity between the predicted cell atlas and the original cell atlas. Finally, we compared the resulting t-SNE embeddings against the ground-truth cell-type labels.

### 2.7 Case study #5

In this case study, massively parallel reporter assay (MPRA) data of a synthetic cAMP-regulated enhancer in human cells were download from GSE31982. We constructed a machine learning dataset correlating approximately 27 000 distinct enhancer variants with their log-transformed activities, subsequently partitioning the data randomly into training and test sets. For the general experimental settings, we employed the baseline model architecture and same training procedures as described above. Following the evaluation of predictive performance, we applied in silico mutagenesis (ISM) to systematically mutate each sequence position and quantify the corresponding effects on predicted activity. By visualizing these mutational profiles, we dissected and analysed the underlying functional regulatory elements. Furthermore, within exploratory design modules, we leveraged the trained MPRA predictor as an in silico objective function to drive gradient-ascent sequence design and greedy ISM refinement.

## 3 Results

### 3.1 Overview of DeepGeSeq

**Figure 1:**
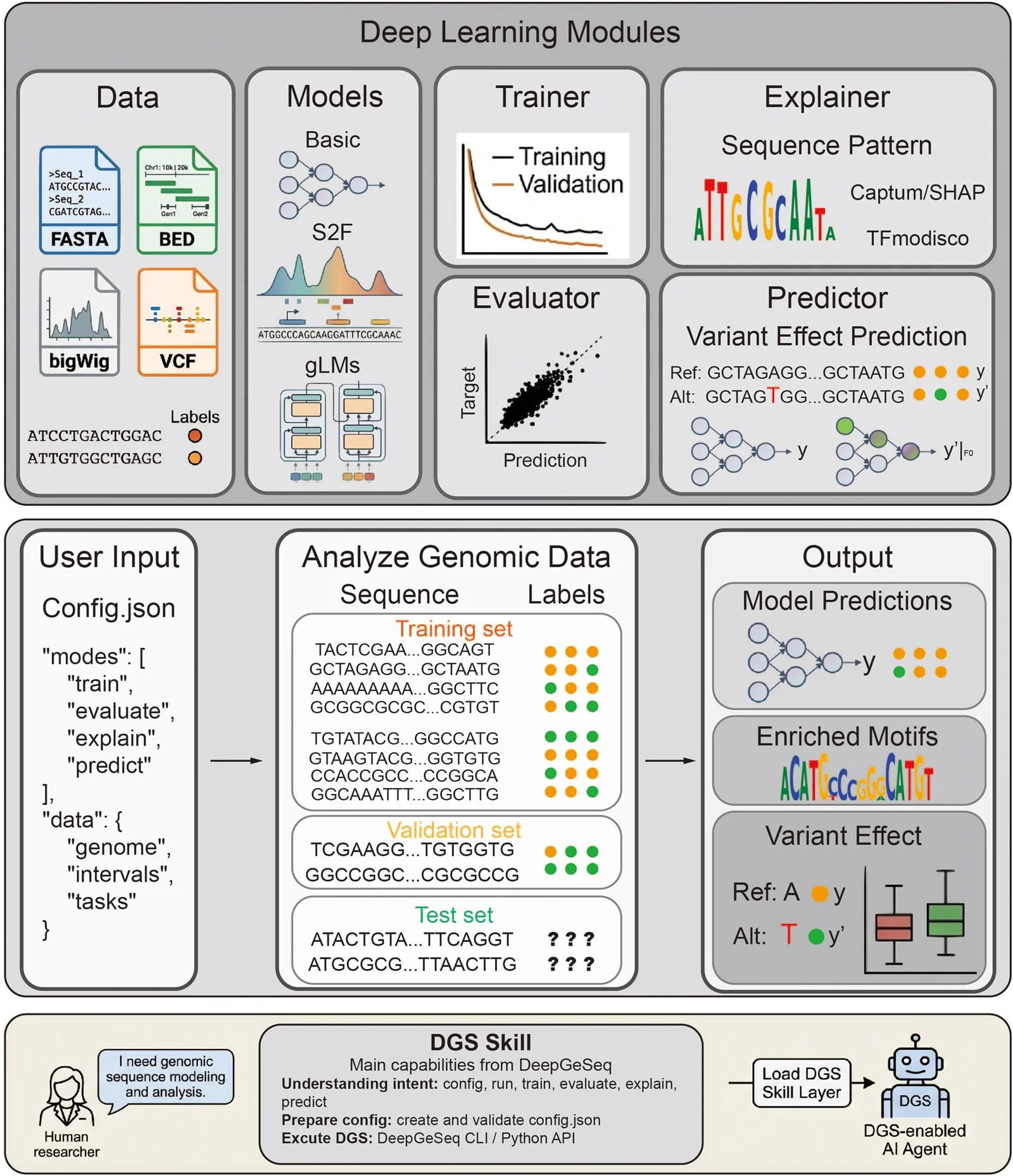
Overview of the DeepGeSeq architecture and workflow. The framework is composed of three interconnected layers: (Top) Deep Learning Modules. DeepGeSeq provides a comprehensive library implemented in PyTorch, featuring a Data module capable of processing standard genomic data (e.g. FASTA, BED, bigWig, and VCF), diverse model architectures (e.g. basic CNNs, published models, and gLMs), Trainer/Evaluator for model optimization and performance evaluation, and an Explainer/Predictor suite to extract learned sequence motifs and score variant effects. (Middle) Automated Analytical Workflow. Driven by a simple JSON configuration file, the pipeline seamlessly processes curated datasets, which are partitioned into training, validation, and test sets of sequence-label pairs, to automatically execute training, evaluation, and prediction tasks. This workflow ultimately yields model predictions, enriched motifs, and variant effect quantifications. (Bottom) Client and Agentic Interface. To further lower the technical barrier, DeepGeSeq integrates a Client and Skill layer, empowering researchers to configure and execute complex genomic sequence modeling tasks entirely through natural language interactions with a DGS-enabled AI agent.

We implemented DeepGeSeq to facilitate the easy development, training, interpretation, and analysis of powerful deep learning models for various genomics data (Fig. 1). DeepGeSeq features: (i) data preprocessing modules that handle DNA sequences, genomic intervals, target signals, and mutation records in FASTA, BED, bigWig, and VCF file formats; (ii) state-of-the-art deep learning modules [e.g. residual mechanism (He *et al.* 2016), attention mechanism (Vaswani *et al.* 2017), and multi-task learning framework (Ruder 2017)], along with ready-to-use model architectures, including binary and regression models (e.g. CNN, DeepSEA, and Basset), profile-based models (e.g. ChromBPNet, Borzoi, and Enformer), and genomic language models (e.g. Evo, DNABERT, and NT models); (iii) automated pipelines for training, predicting, and evaluating the instantiated models; and (iv) deep learning model-based explainer and predictor modules, enabling sequence motif interpreting, which identifies and converts model-activated sequence patterns into biological motifs, and variant effect prediction methods, which calculate the predicted differences between alternative and native sequences.

The deep learning pipeline for sequence analysis is exemplified as follows (Fig. 1). First, users provide reference FASTA sequences, genomic intervals, BED or bigWig targets, a model specification, and an output directory through either a JSON configuration file or the Python API. DeepGeSeq then automatically constructs the datasets, trains the model parameters, and evaluates the predictive performance of the trained model on the test set. After obtaining a well-trained deep learning model, DeepGeSeq provides a suite of tools for deep learning-based sequence analysis, including in silico mutagenesis, sequence motif extraction, and variant effect prediction.

We also explicitly designed DeepGeSeq to be a user-friendly command-line tool and an agentic skill, manageable via a simple configuration file (Fig. 1). Importantly, a corresponding DGS skill was developed, allowing biologists to interact with the AI agent and the DeepGeSeq framework using natural language. For example, in common functional genomics experiments, the primary outputs of upstream pipelines typically comprise BED, bigWig, or VCF files. Users can set the JSON configuration either by manual entry or by interacting with the DGS-enabled AI agent. By default, DeepGeSeq can directly process these input files to automatically train and evaluate the deep learning model, and subsequently utilize the trained model to produce enriched sequence motifs or score variant effects for downstream analysis. Detailed pipeline usage and examples are provided in the tutorials of DeepGeSeq.

### 3.2 Case studies

To demonstrate and verify the efficacy of DeepGeSeq, we conducted a series of case studies. Initially, we (i) validated the deep learning framework using a synthetic dataset to ensure its robustness. Subsequently, we leveraged a published architecture to (ii) reproduce and enhance a previously reported model on real-world data, and (iii) utilize the trained model for the analysis of genomic sequences. Additionally, we extended the application of the deep learning framework to domain-specific data, including (iv) single-cell datasets and (v) MPRA datasets, further validating the framework’s utility in diverse genomic studies. All the case studies were also provided as the tutorials of DeepGeSeq.

#### 3.2.1 Validate the deep-learning framework on synthetic dataset

**Figure 2:**
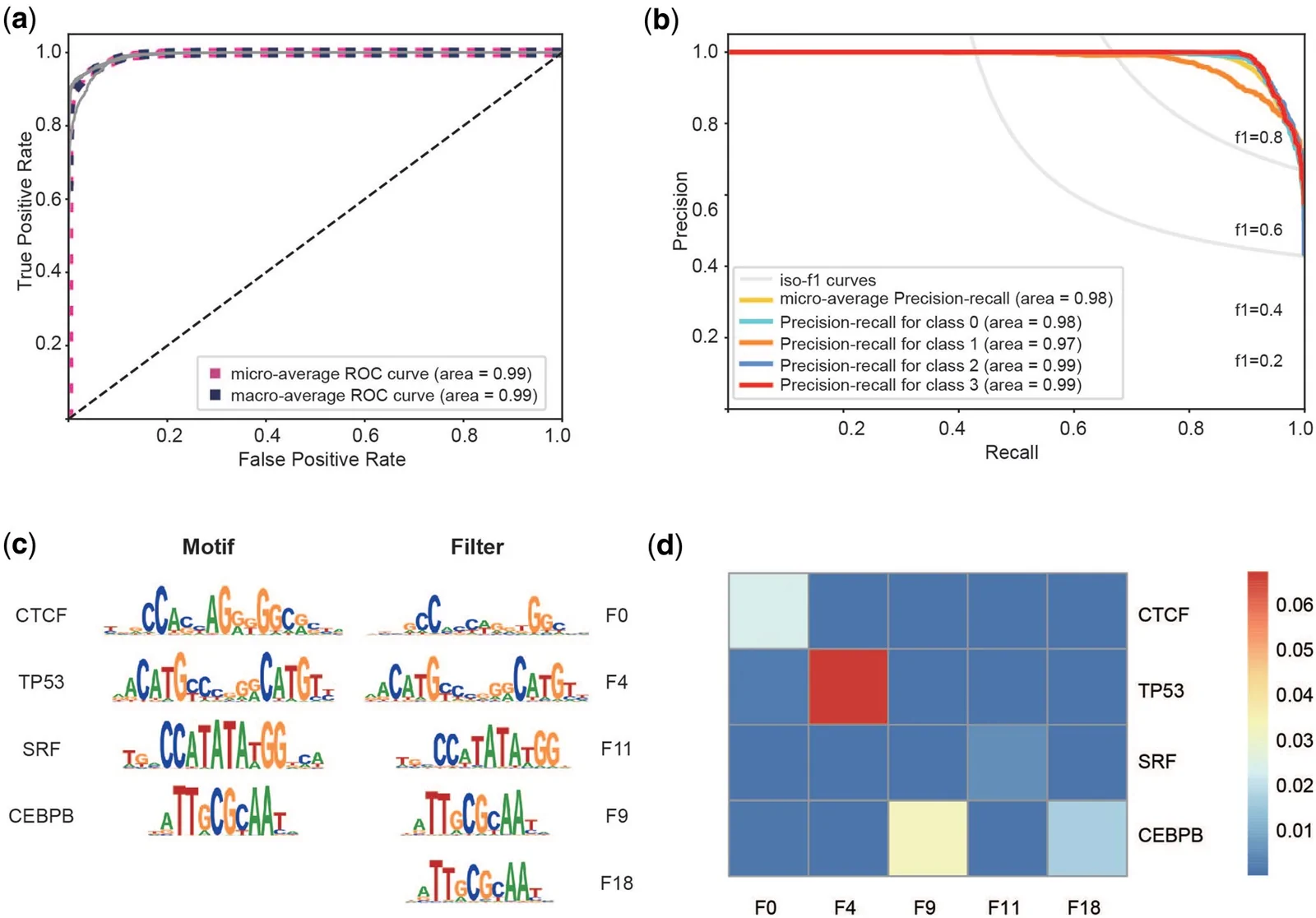
Validation of the DeepGeSeq framework on the synthetic dataset. (a, b) Predictive performance measured by the AUROC (a) and AUPR (b) metrics on the held-out test set. (c) Visualization of ground-truth motifs compared against the sequence patterns extracted by DeepGeSeq. (d) Heatmap of individual filter contributions to motif classification using gradient-based interpretation methods.

The first case study is to demonstrate and validate the deep learning model on a synthetic dataset with minimal effort. In this experiment, we first generated a synthetic dataset by inserting ground truth motifs into random DNA sequences. Specifically, CTCF, TP53, SRF, and CEBPB motifs were embedded in the synthetic sequences to generate a multitask classification dataset. Then, we initialized a baseline CNN model in DeepGeSeq, with the numbers of filters (*N*) set to 20, a filter length (*L*) of 30, and a pooling size (*P*) of 30 in the first convolutional layer to capture the full motifs. All the parameters configuration can be set in a JSON file without requiring any complicated code experience. DeepGeSeq can then automatically perform the deep learning experiment, including model training, validation and interpretation. The CNN model trained on the synthetic dataset achieved an average AUROC of 0.9930 (Fig. 2a) and AUPR of 0.9823 (Fig. 2b) on the held-out test-set.

Having trained and validated the deep learning model on the synthetic genomic dataset, we sought to interpret the sequence patterns that the model represented internally for its accurate prediction. Using the DeepGeSeq’s feature-map-based and TF-MoDISco-based motif extraction, we found that first-layer filters successfully captured the ground truth motifs. For example, the sequence patterns of Filter0 (F0), Filter4 (F4), and Filter11 (F11) faithfully recaptured the canonical motifs of CTCF, TP53, and SRF, respectively (Fig. 2c). Furthermore, we applied DeepGeSeq’s gradient-based and SHAP-based methods to query the filter contributions to different motif classifications. The results showed that those filters that captured the motif representation contributed most to the prediction of the corresponding classification tasks (Fig. 2d). For example, F0, F4, and F11, which were shown to be the motif representations of CTCF, TP53, and SRF, also highly contributed to the prediction of the corresponding TFBS (Fig. 2c and d). Notably, two filters, F9 and F18, both detected the sequence pattern of CEBPB and contributed to the prediction of CEBPB, highlighting the feature redundancy inherent in convolutional neural networks.

#### 3.2.2 Reproduce and apply a published model to real-world data

In the second case study, we utilized DeepGeSeq to reproduce DeepSEA, a pioneering deep learning framework that established a comprehensive approach for learning the regulatory sequence code from large-scale chromatin-profiling data and predicting the functional effects of variants. Specifically, DeepSEA leveraged a dataset of 919 diverse genome-wide chromatin profiles derived from the ENCODE and Roadmap Epigenomics projects, encompassing transcription factor (TF) binding, DNase I sensitivity, and histone-mark profiles. The original study introduced a deep convolutional neural network architecture designed to efficiently scan the sequence patterns and represent the regulatory code. Thereby, DeepSEA significantly improved the prioritization of functional variants, including expression quantitative trait loci (eQTLs), phenotype-associated SNPs, and disease-associated variants from genome-wide association studies (GWAS).

**Figure 3:**
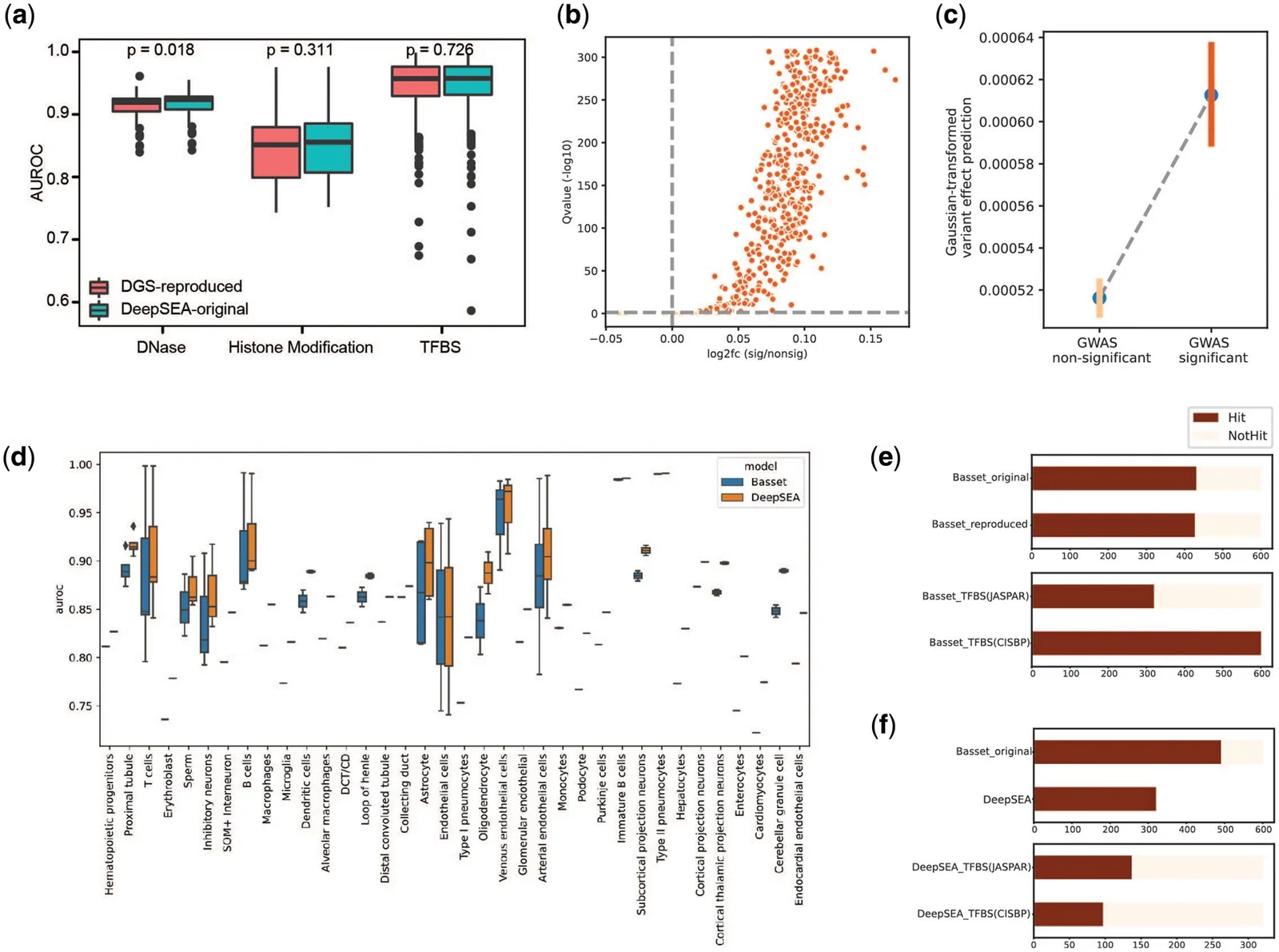
Demonstration of DeepGeSeq for the reproduction and deployment of published models. (a) Boxplot comparing the AUROC scores of the DeepGeSeq-reproduced DeepSEA model with the originally reported architecture on the chromatin dataset. (b) Statistical comparison revealing that a total of 669 out of 690 TFBS genomic features exhibit a significant difference between the disease-associated and non-significant variants (one-sided Wilcoxon rank-sum test). (c) Visualization of the genomic feature with the most significant difference, depicting the mean and 95% confidence intervals of the quantile-normalized (against the Gaussian distribution) between two variants groups. (d) Boxplot showing the AUROC scores of the DeepGeSeq-reproduced Basset and DeepSEA models evaluated on the mouse sciATAC dataset, stratified by cell types. (e, f) Barplots summarizing the annotation of motifs derived from the Basset (e) and DeepSEA (f) models, compared against originally reported motifs and known TFBS motifs (JASPAR and CISBP). “Hit” indicates accurately matched motifs, whereas “NotHit” indicates mismatched motifs identified by TomTom.

Here, we used DeepGeSeq to retrain the DeepSEA architecture on the original dataset, and evaluated the performance of our reproduced model against the metrics reported in the original study. Utilizing the identical held-out test set for a fair comparison, the evaluation demonstrated that the DeepSEA model reproduced by DeepGeSeq achieved performance metrics highly comparable to those reported in the original publication. Specifically, the reproduced model exhibited an average AUROC of 0.9303 and AUPR of 0.9433 across all 919 chromatin tasks (Fig. 3a and Supplementary Fig. 1). For TF binding sites, the median AUROC reached 0.9572, while DNase I sensitivity and histone-mark predictions achieved median AUROC scores of 0.9194 and 0.8514, respectively.

We further applied variant effect prediction to evaluate DeepGeSeq’s ability to prioritize functionally relevant genetic variants. DeepGeSeq quantifies the differential impact of alternative alleles relative to native reference sequences by leveraging the sequence-based model’s capacity to predict the chromatin effects of sequence alterations. To demonstrate this capability, we performed variant effect prediction on 1 06 87 126 genetic variants, including 4 22 398 significant variants (*P* < .05) and 3 842 725 non-significant variants (*P* > .50), derived from an Alzheimer’s disease meta-analysis study (Schwartzentruber *et al.* 2021). We then compared the predicted alteration effects in the TFBS features between two variant sets using the one-sided Wilcoxon rank-sum test with Benjamini-Hochberg correction. Specifically, the predicted variant effects for the significant variant set were significantly higher than those for the non-significant variant set across 669 out of 690 TFBS features (Fig. 3b). Notably, using the most statistically significant TFBS feature, the variant effect predictions generated by the DeepSEA model demonstrated a clear ability to distinguish disease-associated variants from non-significant background variants (Fig. 3c).

#### 3.2.3 Fine-tune and interpret published models on user-provided data

In addition to training on original datasets, we further demonstrated the versatility of DeepGeSeq by applying it to train published model architectures on user-provided datasets. This capability addresses a critical need for researchers who aim to utilize deep learning for practical applications rather than focusing solely on developing novel architectures. In this case study, we showcased this common scenario by training the Basset and DeepSEA architectures on an *in vivo* single-cell chromatin accessibility dataset from sci-ATAC-seq (Cusanovich *et al.* 2018). Specifically, Basset is a convolutional genomics deep learning model architecture similar to DeepSEA, which can be applied to regulatory sequence modeling and analysis. The sci-ATAC dataset encompasses chromatin accessibility profiles across 13 tissues in adult mice, providing a rich resource for studying cell-type-specific chromatin accessibility and regulatory elements. In the original research article, researchers trained the Basset model on the sci-ATAC dataset and investigated the associated transcription factor binding motifs underlying *in vivo* chromatin accessibility by interpreting the de novo motifs derived from the Basset model.

Here, we used DeepGeSeq to retrain the Basset and DeepSEA model architectures on the mouse sci-ATAC dataset, and compared the learned transcription factor motifs with the reported results. Because the original paper did not report the prediction performance, we compared the performance between Basset and DeepSEA using the same test dataset. After training, the Basset architecture reached an average AUROC of 0.8597 and AUPR of 0.1400 on the sci-ATAC1 dataset across 85 cell-types, while DeepSEA achieved the average AUROC of 0.8873 and AUPR of 0.1518 (Fig. 3c and Supplementary Fig. 2).

We used the DeepGeSeq’s feature-map-based explaination methods to extract motifs and generate the position frequency matrix (PFM) for each filter in the first convolutional layer. Specifically, we extracted 600 motifs with a length of 19 from the Basset model, which is identical to the original study, and 320 motifs with length of 8 from the DeepSEA model, as defined by their respecitive hyperparameters. We then compared the motifs reproduced by Basset and DeepSEA against the originally reported motifs. The results showed that our reproduced Basset model recaptured 427 out of 600 motifs reported in the original study (Fig. 3d). We also found that all 320 motifs from the DeepSEA model were identical to the originally reported Basset motifs, while 109 out of 600 reported Basset motifs could not be recaptured by the DeepSEA model (Fig. 3e). Furthermore, we annotated the Basset- and DeepSEA-derived motifs against the JASPAR and CIS-BP databases using TomTom algorithm (Gupta *et al.* 2007). For the Basset model, 320 and 600 de novo motifs were successfully assigned to known TFBS in JASPAR (Rauluseviciute *et al.* 2024) and CIS-BP (Weirauch *et al.* 2014) database, respectively (Fig. 3d). Similarly, for the DeepSEA model, 137 and 97 de novo motifs were assigned to known TFBS in the JASPAR and CIS-BP databases, respectively (Fig. 3e).

#### 3.2.4 Apply the deep-learning framework on single-cell dataset

The functional regulatory genome is dynamically activated across spatial and temporal contexts, reflecting the intricate orchestration of gene regulation. Single-cell Assay for Transposase Accessible Chromatin sequencing (scATAC-seq) enables the characterization of chromatin accessibility heterogeneity at the single-cell level, offering unprecedented insights into the regulatory genome. In this case study, we leveraged the power of deep learning to predict chromatin accessibility at the single-cell resolution and generate low-dimensional embeddings of individual cells, demonstrating DeepGeSeq’s utility in domain-specific applications.

We began by constructing a machine learning dataset using a widely benchmarked scATAC dataset of hematopoietic differentiation (Buenrostro *et al.* 2015). This dataset paired DNA sequences with chromatin accessibility profiles for each peak across individual cells. We utilized DeepGeSeq-derived architectures to develop a deep learning model for predicting chromatin accessibility at single-cell resolution. Specifically, we employed Convolutional Neural Networks (CNNs) to extract sequence patterns underlying each genomic peaks and incorporated Multi-task Learning (MTL) to jointly predict chromatin accessibility across all single cells.

**Figure 4:**
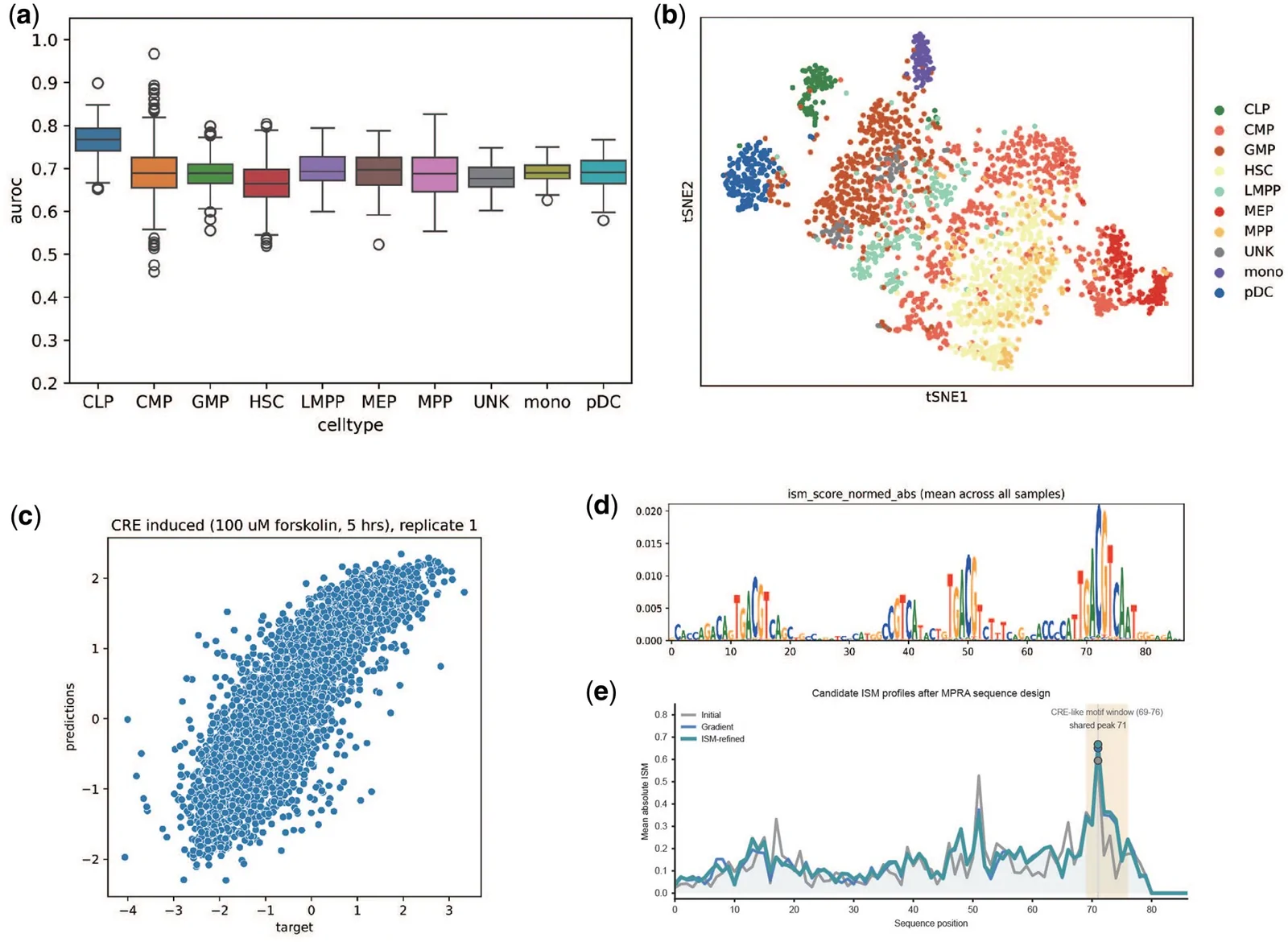
Application of the deep-learning framework on single-cell and MPRA datasets. (a) Boxplot detailing the predictive performance on held-out peaks for the scATAC dataset, evaluated by AUROC scores with cells grouped by cell types. (b) t-SNE visualization of cell representations derived from the weights of the final classifier layer, colored by cell type. The cell type labels refer to hematopoietic stem cell (HSC), multipotent progenitor (MPP), lymphoid primed MPP (LMPP), common lymphoid progenitor (CLP), plasmacytoid dendritic cell (pDC), common myeloid progenitor (CMP), granulocyte macrophage progenitor (GMP), megakaryocyte-erythroid progenitor (MEP) cell, monocyte (Mono) and unknown (UNK). (c) Scatterplot showing experimental observed and predicted mutagenesis effects for the cAMP-responsive enhancer. (d) Visual representations of the sequence architecture of the CRE by ISM analysis. (e) ISM profiles of in silico designed sequence. The line plot visualizes the mean absolute in silico mutagenesis (ISM) scores across sequence positions for the initial seed (grey), gradient-ascent optimized (blue), and ISM-refined (teal) candidates. A shaded region highlights the shifted CRE-like motif window (positions 69–76).

After training, the model demonstrated robust performance, achieving a mean AUROC score of 0.6890 when evaluated across peaks per cell, and an average AUROC score of 0.7101 when evaluated across cells per peak. The AUROC scores by cell type confirmed the model’s ability to effectively predict chromatin accessibility patterns across all tested cell types (Fig. 4a and Supplementary Fig. 3). Beyond training and evaluating the model, we also explored important downstream applications, such as cell embedding and visualization for single-cell analysis. Specifically, we generated low-dimensional embeddings of individual cells using the learned weight matrix from the final layer of the MTL model. The resulting t-SNE projections revealed that cells of the same type spontaneously formed distinct clusters corresponding to expert-annotated cell-type labels (Fig. 4b), underscoring the model’s effectiveness in resolving cellular heterogeneity.

#### 3.2.5 Apply the deep learning framework on MPRA dataset

The Massively Parallel Reporter Assay (MPRA) is a powerful tool for systematically quantifying the relative activities of synthetic regulatory element libraries, thereby providing a comprehensive dissection of the sequence landscape of transcriptional regulatory elements. In our final case study, we employed MPRA data to train quantitative sequence-activity models and utilized in silico mutagenesis (ISM) for a systematic analysis of transcriptional regulatory elements.

Leveraging MPRA data detailing the activities of a synthetic cAMP-regulated enhancer in human cells (Melnikov *et al.* 2012), we constructed a machine learning dataset by correlating approximately 27 000 distinct random enhancer variants with their corresponding log-transformed activities. We utilized DeepGeSeq to develop a quantitative sequence-activity model based on the CNN architecture. After training, the model achieved a Pearson correlation coefficient (PCC) score of 0.84 and a Spearman correlation coefficient (SCC) score of 0.85 when comparing predicted and observed activities on the held-out dataset (Fig. 4c). Subsequently, we applied ISM analysis using the trained model to systematically elucidate the architecture of the cAMP-responsive enhancer. Specifically, ISM involves converting every position in the sequence to every other possible base and quantifying the effects of these mutations using the predictive model. By visualizing the predicted absolute fold-change of mutated sequences relative to the original sequence (Fig. 4d), we identified the footprints of the CREB motif (consensus TGACGTGCA) within this enhancer, which is consistent with previously reported results.

Beyond sequence interpretation, we further leveraged the trained sequence-activity model to perform exploratory in silico sequence design. This workflow substantially increased the enhancer activity prediction from the seed sequence after gradient-ascent design and greedy ISM refinement. Motif scanning of the designed sequences revealed that a CREB-like motif was retained with the best match in the position 69–76 window in the gradient-designed and ISM-refined sequences (Fig. 4e). Collectively, these validation steps establish the utility of DeepGeSeq for exploratory sequence design and diagnostics, yielding promising candidates for further experimental validation.

## 4 Discussion

In summary, DeepGeSeq is a versatile and user-friendly deep learning framework designed to streamline genomic sequence analysis. It offers an comprehensive end-to-end workflow encompassing multi-omics data processing, state-of-the-art model architectures, automated training pipelines, and advanced biological interpretation. By integrating flexible configuration files and agentic skills for natural language interaction, DeepGeSeq bridges the gap between complex computational techniques and practical usability, facilitating broader adoption in biological research. Furthermore, our case studies illustrate how DeepGeSeq can be effectively leveraged for advanced sequence analysis. Collectively, these applications highlight the robustness and reproducibility of DeepGeSeq’s workflows, as well as the capacity of deep learning to model regulatory genome and capture complex sequence patterns.

Beyond specific neural network architectures, the field of computational genomics is currently undergoing a paradigm shift towards large-scale foundation models, widely referred to genomic language models (gLMs). Pre-trained on massive corpora of diverse genomic sequences, gLMs have demonstrated unprecedented capabilities in deciphering complex sequence syntax and generating regulatory elements. Recognizing this trajectory, DeepGeSeq’s highly modular design intrinsically serves as a forward-looking interface for these foundation models. In future iterations, we plan to seamlessly integrate more pre-trained gLMs into the DeepGeSeq ecosystem, enabling users to perform downstream tasks, such as single-cell resolution modeling and in silico regulatory sequence design, with minimal configuration overhead.

To lower the technical barrier, DeepGeSeq integrates a highly modular architecture with a user-friendly interface and specialized agentic skills. By seamlessly combining these modular components with an AI agent, we could transform DeepGeSeq into an intuitive tool that enables biologists to analyse genomic sequences and configure deep learning workflows entirely through natural language. This natural language interface elegantly bridges the gap between complex deep learning models and task-specific biological applications. Ultimately, DeepGeSeq serves as a vital conduit, empowering researchers to harness the full potential of AI models for highly customized regulatory discoveries.

## Supplementary Material

btag584_Supplementary_Data

## Data Availability

Code and datasets are available at https://github.com/JiaqiLi1024/DeepGeSeq under DOI: 10.5281/zenodo.21333209.
