# Supplementary material for: DeepGeSeq: deep learning library for genomic sequence modeling and analysis
Source: Bioinformatics. 2026 Aug 4;42(8):btag584. doi: 10.1093/bioinformatics/btag584 (PMC13481688; doi:10.1093/bioinformatics/btag584)
Supplement: Supplementary file 1 [file btag584_supplementary_data.pdf]

Supplementary Figure

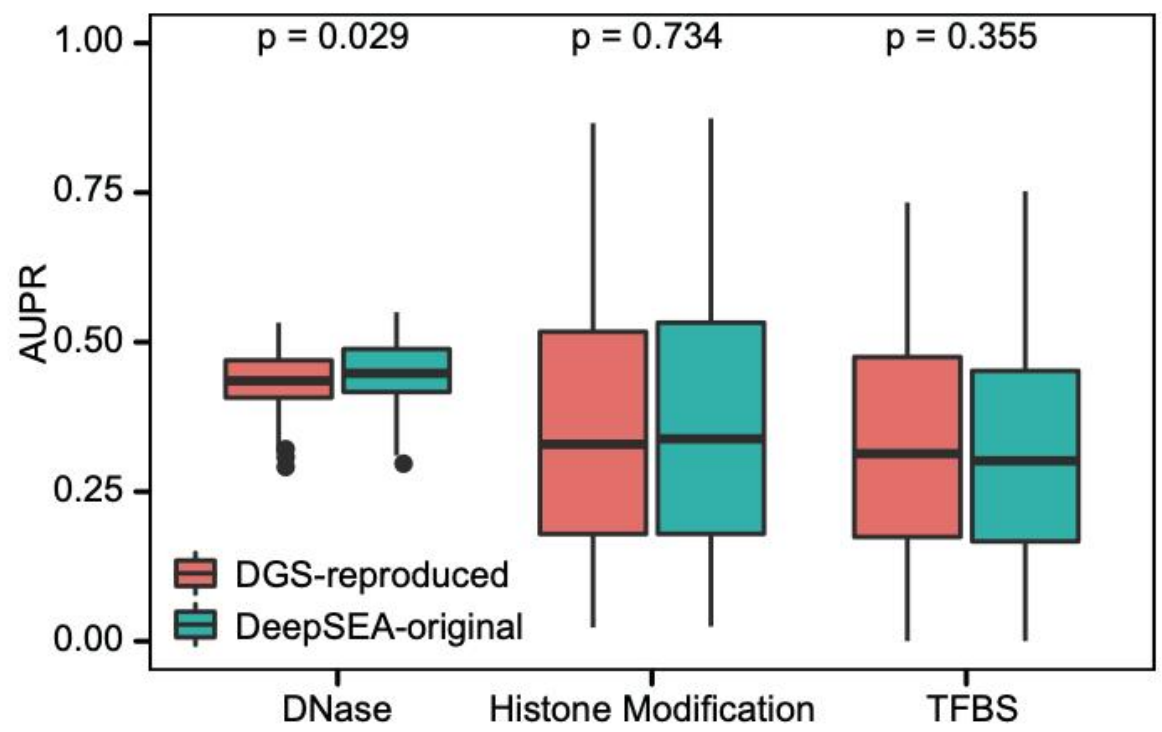

SF 1. Boxplot showing the AUPR scores of the DeepGeSeq-reproduced and previously reported DeepSEA architecture on the chromatin dataset.

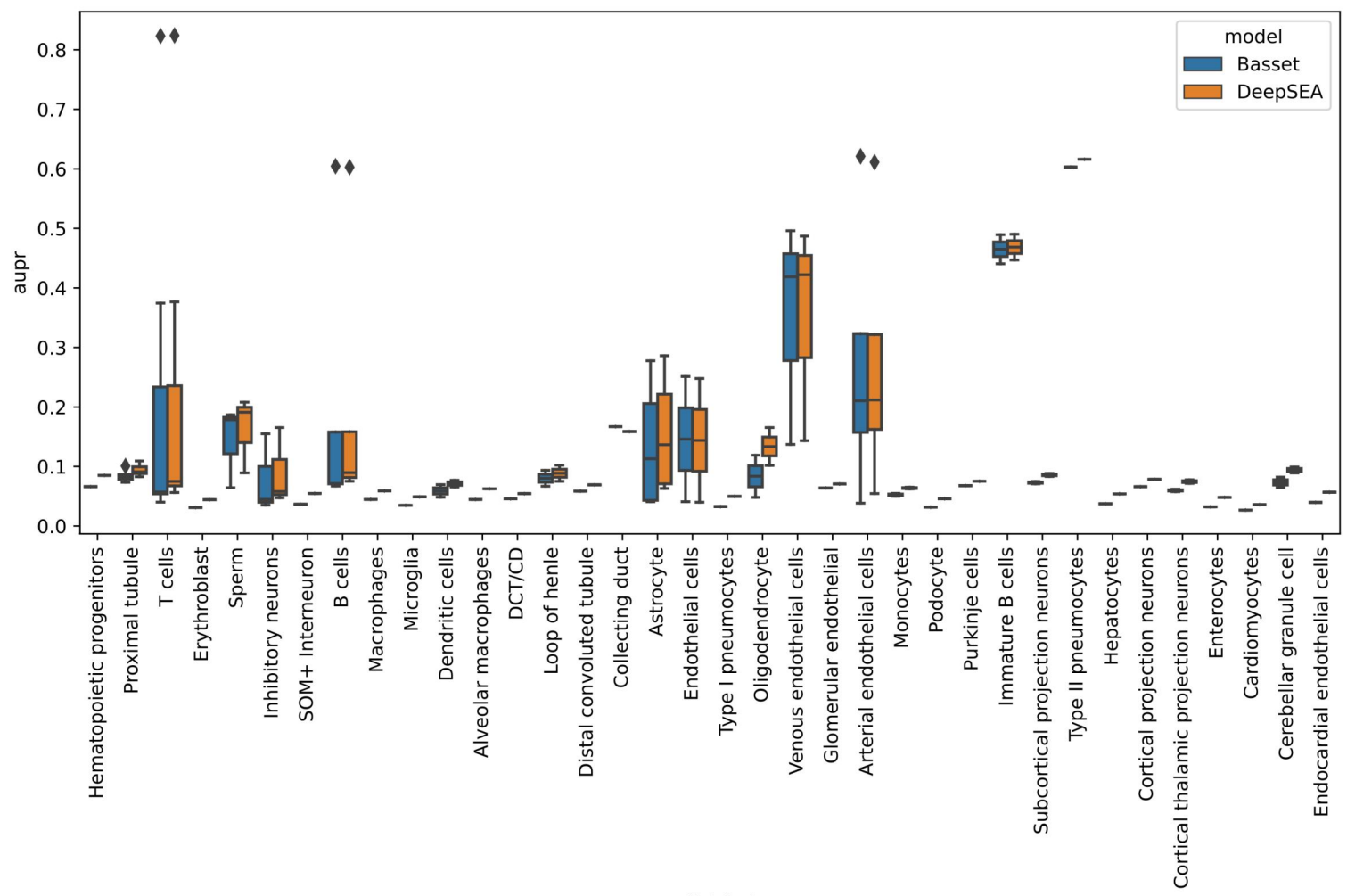

SF 2. Boxplot detailing the AUPR scores of the DeepGeSeq-reproduced Basset and DeepSEA models on the mouse sciATAC dataset.

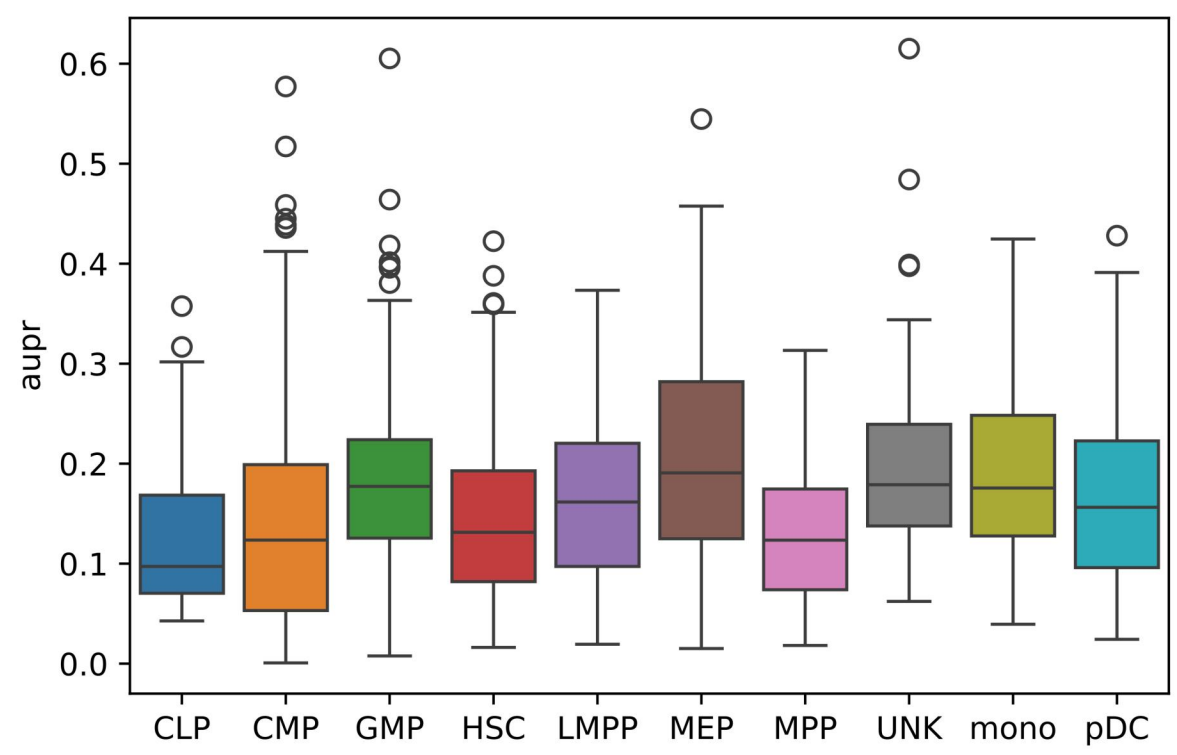

SF 3. Boxplot of the predictive performance on held-out peaks for the scATAC dataset, evaluated by AUPR scores grouped by cell types.

Table 1: Comparison of DeepGeSeq with existing deep learning frameworks for genomics

Feature	DeepGeSeq (This Work)	Kipoi	Selene	Janggu	EUGENe	gReLU
**Year introduced**	2026	2019	2019	2020	2023	2024
**End-to-End Workflow**	Yes (From data processing to interpretation)	No (Focuses on pre-trained model distribution)	Yes (Focuses on model training pipelines)	Yes (Focuses on data acquisition, evaluation)	Yes (From ETL to evaluation & interpretation)	Limited (Focuses on model architecture design)
**Configuration Usability**	High (User-friendly JSON configuration, no coding required)	Low (Requires basic CLI/Python)	Moderate (Requires YAML/Python scripting)	Low (Requires Python scripting)	Moderate (Supports YAML configuration and Python API)	Low (Requires Python coding expertise)
**Agentic Skill**	Yes (Interact AI Agent with natural language)	No	No	No	No	No
**Model Architectures**	Wide range of pre-trained models, gLMs	Wide range of pre-trained models	PyTorch-based custom architectures	Keras-based custom architectures	PyTorch-based custom architectures	PyTorch-based custom architectures
**Variant Effect Prediction**	Yes (*In silico* mutagenesis & scoring)	Limited (Via separate plugins/wrappers)	Yes (*In silico* mutagenesis & scoring)	Limited (Leverages higher-order sequence encoding)	Yes (*In silico* saturation mutagenesis)	Yes (With data augmentation & statistical testing)
**Model Interpretation**	Yes (Built-in Feature-map, DeepLIFT/SHAP, TF-MoDISco)	Limited (Via separate plugins)	Limited (*In silico* mutagenesis visualization)	Yes (Built-in Integrated Gradients)	Yes (SeqExplainer: ISM, DeepLIFT, GradientSHAP, TF-MoDISco)	Yes (DeepLIFT/SHAP, TF-MoDISco)
**Single-Cell Data Support**	Yes (Built-in support for scATAC-seq modeling & clustering)	No	No	No	No native support (Currently planned for future)	Limited (Supports pseudobulk scATAC-seq)
